# Predicted Peptides from Non-Structural Proteins of Porcine Reproductive and Respiratory Syndrome Virus Are Able to Induce IFN-γ and IL-10

**DOI:** 10.3390/v5020663

**Published:** 2013-02-11

**Authors:** Alexel Burgara-Estrella, Ivan Díaz, Irene M. Rodríguez-Gómez, Sabine E. Essler, Jesús Hernández, Enric Mateu

**Affiliations:** 1 Laboratorio de Inmunología, Centro de Investigación en Alimentación y Desarrollo A.C (CIAD), Carretera a la Victoria Km 0.6, C.P. 83304 Hermosillo, Sonora, Mexico;E-Mail: alexel_25@hotmail.com (A.B.-E.); 2 Centre de Recerca en Sanitat Animal (CReSA), UAB-IRTA, Campus de la Universitat Autònoma de Barcelona, 08193 Bellaterra, Barcelona, Spain; E-Mail: Ivan.Diaz@cresa.uab.cat (I.D.); irenero22@hotmail.com (I.M.R.-G.); 3 Departamento de Anatomía y Anatomía Patológica Comparadas, Facultad de Veterinaria, Universidad de Córdoba, Córdoba, Spain; 4 Department of Pathobiology, Institute of Immunology, University of Veterinary Medicine Vienna, Vienna, 1210, Austria; E-Mail: sabine.essler@phylo-dat.net (S.E.E.); 5 Departament de Sanitat i d’Anatomia Animals, Universitat Autònoma de Barcelona, 08193 Bellaterra, Barcelona, Spain

**Keywords:** PRRSV, non-structural proteins, epitopes, interferon gamma, IL-10

## Abstract

This work describes peptides from non-structural proteins (nsp) of porcine reproductive and respiratory syndrome virus (PRRSV) predicted as potential T cell epitopes by bioinfornatics and tested for their ability to induce IFN-γ and IL-10 responses. Pigs immunized with either genotype 1 or genotype 2 PRRSV attenuated vaccines (n=5/group) and unvaccinated pigs (n = 4) were used to test the peptides. Swine leukocyte antigen haplotype of each pig was also determined. Pigs were initially screened for IFN-γ responses (ELISPOT) and three peptides were identified; two of them in non-conserved segments of nsp2 and nsp5 and the other in a conserved region of nsp5 peptide. Then, peptides were screened for IL-10 inducing properties. Six peptides were found to induce IL-10 release in PBMC and some of them were also able to inhibit IFN-γ responses on PHA-stimulated cells. Interestingly, the IFN-γ low responder pigs against PRRSV were mostly homozygous for their SLA haplotypes. In conclusion, these results indicate that nsp of PRRSV contain T-cell epitopes inducing IFN-γ responses as well as IL-10 inducing segments with inhibitory capabilities.

## 1. Introduction

Porcine reproductive and respiratory syndrome (PRRS) is one of the most costly diseases of swine [1]. This syndrome is caused by PRRS virus (PRRSV), that belongs to the family *Arteriviridae* in the order *Nidovirales* and comprises two different genotypes named type 1 (formerly called European) and type 2 (formerly called North-American) [2,3]. PRRSV has a positive-stranded RNA genome of about 15 Kb organized in 10 open reading frames (ORF). ORF1a and 1b encode for two polyproteins that after enzymatic cleavage will result in 14 non-structural proteins (nsp) involved in viral replication. ORF2-ORF7 encode the structural proteins of the virus [4,5]. Non-structural proteins, particularly nsp2, have been shown to contain some dominant linear B epitopes [6,7,8]. Also, nsp1, nsp2, nsp4 and nsp11 have been related to the inhibition of type I IFNs and nsp2 may be also involved in the viral regulation of TNF-α responses of the host cell [9,10]. In PRRSV infection, IL-10 seems to be up-regulated in PBMC and dendritic cells [11,12] and its expression could be inversely related to IFN-γ responses [13]. These inhibitory effects have been related to the ability of PRRSV to evade the immune response of pigs. Structural proteins contain neutralization epitopes that have been reported to exist in glycoproteins (GP) 2, 3, 4, 5 and protein M although the role of GP5 in the induction of neutralizing antibodies is debated nowadays [14,15,16]. 

PRRSV control has proven to be difficult, among other causes because immunity against one strain does not preclude protective immunity against a different one [17,18,19]. The ideal of a universally effective vaccine, namely, one that could protect against all existing PRRSV strains, is far from reach. Broadly reactive T- and B- epitopes that could elicit T-cell responses and neutralizing antibodies against all, or at least a majority of PRRSV strains have not been clearly identified. Identification of such epitopes is therefore a key element for the development of PRRSV vaccinology.

IFN-γ responses have been related with clearing of PRRSV infection [20,21]; therefore, it is important to find relevant regions of the virus that induce IFN-γ responses. In the past, some epitopes inducing IFN-γ have been described among structural proteins [22,23,24]. At the moment, there is only one paper reporting the role of nsp (nsp9 and nsp10) in the induction of T-cell responses [25]. Bioinformatic prediction is a method to screen for potentially relevant T-cell epitopes based on binding to major histocompatibility complex (MHC)-I or MHC-II. Selected peptides have to be tested then by methods such as the IFN-γ ELISPOT or others to corroborate the prediction. In spite of its limitations compared with other methods—for example evaluation of overlapping peptides—prediction allows the detection of immunodominant T-cell epitopes from several proteins in an easy and cheap way. In this study we used bioinformatics for the screening of immunodominant T-cells epitopes in conserved and non-conserved segments of nsp of genotype 1 and 2 of PRRSV. The manuscript reports that certain peptides within nsp of PRRSV may induce IFN-γ responses in PBMC while others induce recall or natural IL-10 release. The ability of the identified IL-10 inducing peptides for inhibiting IFN-γ responses in naïve and PRRS vaccinated pigs was examined.

## 2. Results and Discussion

### 2.1. Peptide-Induced IFN-γ Producing Cells

Usually, variation in nsp is limited because of the essential nature of their functionalities for viral replication. T-epitopes present in conserved regions of nsp would be therefore excellent candidates for a “universal” vaccine. Recently it was reported that peptides of nsp9 and nsp10 of PRRSV were able to induce IFN-γ recall responses [25]. In the present work we screened conserved or non-conserved regions of nsp2, nsp3, nsp5, nsp9 nsp10 of PRRSV for potential T-cell epitopes by using bioinformatics. The examined peptides were composed by: a) the 18 best scoring peptides located in conserved regions of nsp for both viral genotypes; b) the six best scoring peptides with conservation values between 80% and 90% and, c) the four best scoring peptides in nsp of each genotype regardless of their degree of conservation (Table 1). 

IFN-γ responses were evaluated in the ELISPOT at 21 and 49 dpv. At 21 dpv IFN-γ responses were very low increasing substantially at 49 dpv; Table 2 shows the results at 49 dpv. Frequencies of IFN-γ secreting cells against conserved peptides of non-structural proteins of PRRSV were poor, while the IFN-γ response against non-conserved peptides with high prediction scores was clearly present. In group I (vaccinated with the genotype 1 vaccine), 3/5 pigs responded consistently to a non-conserved nsp2 peptide (aa 589-597 SLYKLLLEV). Other three peptides (the non-conserved aa 1114-1149 WLFAGVVLL in nsp2, the conserved aa 1929-1937 LLNEILPAV and the non-conserved aa 2025-2033 IIIGGLHTL, both in nsp5) were recognized by 2/5 pigs. For genotype 2 vaccinated pigs, the response was general of lower intensity. IFN-γ frequencies were only observed in 2/5 pigs for two peptides (aa 589-597 SLYKLLLEV) in nsp2 and one conserved in nsp5 (aa 1929-1937 LLNEILPAV). The frequency of IFN-γ in presence of virus was higher in pigs of group I compared with pigs of group II. Unvaccinated pigs did not respond to any peptide or whole virus.

Compared to the use of overlapping peptides, the bioinformatic approach permit to reduce the costs associated to the production of large number of peptides. Certainly, one common criticism to the bioinformatics approach is about sensitivity. When applied to pigs, the lack of predictive tools based on porcine SLA is also a problem. Regarding the first issue, in the present study we included not only the peptides with the best prediction scores within conserved regions of PRRSV nsp but also, the four peptides with the best prediction scores in non-conserved regions of nsp of genotype 1 and 2 PRRSV. This should serve to assess the sensitivity of the method. As shown by the results, the method of prediction was sensitive and accurate enough to allow the identification of peptides able to induce IFN-γ comparable with those reported by others in structural proteins [22,23]. The best peptide identify in this work was classified as non-conserved (aa 589-597 SLYKLLLEV), which led us to suggest that conserved segments of nsp are not very good candidates for a potentially universal PRRSV vaccine; although the role of conserved aa 1929-1937 LLNEILPAV should be further investigated. In any case, it cannot be discarded that other T-epitopes in conserved regions of PRRSV nsp could exist and were not detected by the methodology and techniques used here.

**Table 1:** Peptides predicted as T-cell epitopes of non-structural proteins (nsp) of porcine reproductive and respiratory syndrome virus (PRRSV) analyzed in the present study.

Protein	aa position	Predicted sequence	Conservation ^a^
nsp2	589-597	SLYKLLLEV	No
nsp2	1061-1069	GRFEFLPKM	No
nps2	1141-1149	WLFAGVVLL	No
nsp3	1337-1345	YIWHFLLRL	No
nsp3	1670-1678	AVRRAALTG	Yes
nsp5	1902-1910	VQLLCVFFL	Yes
nsp5	1929-1937	LLNEILPAV	Yes
nsp5	1960-1968	VLMIRLLTA	No
nsp5	2025-2033	IIIGGLHTL	No
nsp5	2046-2054	ILNEVLPAV	Yes
nsp9	3-11	FKLLAASGL	No
nsp9	142-150	QLPYKLYPV	Yes
nsp9	143-151	FVLPGVLRL	Yes
nsp9	246-254	MAGINGQRF	Yes
nsp9	246-254	MAGINGNRF	Yes
nsp9	258-266	VLPGVLRLV	No
nsp9	325-333	TVTPCTLKK	Yes
nsp9	377-385	LGKNKFKEL	Yes
nsp9	430-438	YVLNCCHDL	Yes
nsp9	524-532	NYHWWVEHL	Yes
nsp9	587-595	YYASAAAIL	Yes
nsp9	594-602	ILMDSCACI	Yes
nsp9	1222-1230	YLPSYVLNC	Yes
nsp10	670-678	VPYKPPRTV	Yes
nsp10	716-724	IPYKPPRTV	No
nsp10	718-726	YKPPRTVIM	Yes
nsp10	974-982	ITIDSSQGA	Yes
nsp11	1116-1124	KELAPHWPV	Yes
nsp11	1116-1124	VELAPHWPV	Yes
nsp11	1166-1174	GTPGVVSYY	No
nsp11	1222-1230	YLPDLEAYL	No

aa = amino acid; ^a ^Those peptides with a maximum of one amino acid of difference between the examined sequences were considered as conserved between genotypes.

**Table 2:** Frequencies of IFN-γ secreting cells per 5 × 10^5^ PBMC (ELISPOT) induced by peptides of non-structural proteins of PRRSV. The table shows only peptides resulting in an adjusted count of at least ≥5 spots/well (cut-off of the test) for one or more pigs.

Protein	aa position	Peptide sequence	Group I^a^ (pig nº)					Group II (pig nº)					Group III (pig nº)			
			66	67	68	69	70	61	62	63	64	65	71	72	73	74
nsp2	589-597	SLYKLLLEV	1	14	10	0	9	0	5	9	0	1	0	0	0	0
nsp2	1061-1069	GRFEFLPKM	0	3	0	0	0	0	4	7	3	3	0	0	0	0
nsp2	1141-1149	WLFAGVVLL	2	10	3	0	11	0	13	7	0	0	0	1	0	0
nsp3	1337-1345	YIWHFLLRL	2	5	5	0	3	0	2	5	0	2	0	0	1	0
nsp5	1929-1937	LLNEILPAV	2	8	0	0	7	0	9	17	0	0	0	0	0	0
nsp5	2025-2033	IIIGGLHTL	2	13	5	0	9	0	6	2	0	2	0	0	0	0
nsp5	2046-2054	ILNEVLPAV	2	8	1	0	5	0	0	5	0	2	0	0	1	0
nsp9	246-254	MAGINGNRF	0	8	0	0	0	0	0	0	0	1	2	0	1	0
nsp9	587-595	YYASAAAIL	0	6	0	0	0	0	1	0	0	0	0	0	0	0
nsp11	1116-1124	VELAPHWPV	0	12	0	0	0	0	0	0	0	0	0	0	0	0
nsp11	1116-1124	KELAPHWPV	0	10	3	0	1	0	0	0	0	0	0	0	0	0
nsp11	1166-1174	GTPGVVSYY	0	5	0	0	0	0	0	0	0	0	0	0	0	0
nsp11	1222-1230	YLPDLEAYL	1	2	31	0	0	0	4	7	0	0	0	0	0	0
	Genotype 1 whole virus		42	29	96	0	17	0	10	12	0	2	0	0	0	0
	Genotype 2 whole virus		0	1	6	0	14	5	7	2	0	0	0	0	0	0

**Table 3:** Swine leukocyte antigen (SLA) class I and class II low-resolution (Lr) haplotypes identified by PCR-SSP in pigs included in the present study. Identification numbers 61-70 correspond to vaccinated pigs and numbers pigs 71–74 correspond to naïve unvaccinated controls.

	SLA class I				SLA class II			
Pig n°	SLA-1	SLA-2	SLA-3	Inferred haplotype	DQA	DQB1	DRB1	Inferred haplotype
61	04XX	04XX	04XX/hb06	Lr-04.0	04XX+w05XX	09XX	09XX/La02	Lr-0.27
	11XX (1103)	jh02	05XX	Lr-59.0	02XX/ka01	Blank	06XX	Lr-0.32
62	04XX	01XX	01XX	Lr-LD-01.0	01XX	07XX	06XX	Lr-0.12
	blank	05XX	06XX (0601)	Lr-47.0	03XX	07XX	04XX	Lr-0.19a
63	04XX	04XX	04XX/hb06	Lr-04.0	02XX/ka01	04XX	02XX	Lr-0.04
	13XX	10XX	05XX	Lr-64.0	01XX	06XX/zs12	10XX	Lr-0.23
64	04XX	04XX	04XX/hb06	Lr-04.0	02XX/ka01	04XX	02XX	Lr-0.04
	04XX	04XX	04XX/hb06	Lr-04.0	02XX/ka01	04XX	02XX	Lr-0.04
65	04XX	04XX	04XX/hb06	Lr-04.0	04XX+w05XX	09XX	09XX/La02	Lr-0.27
	11XX (1103)	jh02	05XX	Lr-59.0	02XX/ka01	Blank	06XX	Lr-0.32
66	09XX	05XX	07XX	Lr-28.0	01XX	06XX/zs12	10XX	Lr-0.23
	11XX (1103)	jh02	05XX	Lr-59.0	04XX+w05XX	09XX	09XX/La02	Lr-0.27
67	04XX	04XX	04XX/hb06	Lr-04.0	02XX/ka01	02XX	04XX	Lr-0.15a
	11XX (1103)	jh02	05XX	Lr-59.0	04XX+w05XX	09XX	09XX/La02	Lr-0.27
68	04XX	01XX	01XX	Lr-LD-01.0	01XX	07XX	06XX	Lr-0.12
	blank	05XX	04XX/hb06	Lr-34.0	04XX+w05XX	09XX	13XX	Lr-0.25
69	04XX	04XX	04XX/hb06	Lr-04.0	02XX/ka01	02XX	04XX	Lr-0.15a
	04XX	04XX	04XX/hb06	Lr-04.0	02XX/ka01	02XX	04XX	Lr-0.15a
70	01XX	01XX	01XX	Lr-01.0	01XX	01XX	01XX	Lr-0.01
	11XX (1103)	jh02	05XX	Lr-59.0	04XX+w05XX	09XX	09XX/La02	Lr-0.27
71	04XX	01XX	01XX	Lr-LD-01.0	01XX	07XX	06XX	Lr-0.12
	blank	05XX	04XX/hb06	Lr-34.0	01XX	07XX	06XX	Lr-0.12
72	04XX	04XX	04XX/hb06	Lr-04.0	02XX	04XX	11XX	Lr-0.26
	11XX (1103)	jh02	05XX	Lr-59.0	04XX+w05XX	09XX	09XX/La02	Lr-0.27
73	04XX	04XX	04XX/hb06	Lr-04.0	01XX	06XX/zs12	10XX	Lr-0.23
	11XX (1103)	jh02	05XX	Lr-59.0	04XX+w05XX	09XX	09XX/La02	Lr-0.27
74	04XX	04XX	04XX/hb06	Lr-04.0	01XX	06XX/zs12	10XX	Lr-0.23
	11XX (1103)	jh02	05XX	Lr-59.0	04XX+w05XX	09XX	09XX/La02	Lr-0.27

Parida *et al.* [25] have reported two peptides from nsp9 and two peptides from nps10 as IFN-γ inducers. In our work, frequencies of IFN-γ secreting cells in response of nsp9 and nsp10 were low or nil and, in most cases, did not reach the minimum value (>5 spots) to be considered significant. The reason for these apparently contradicting results may reside firstly in the different approach (overlapping peptides *versus* prediction). Secondly, with the aim of having high specificity, the criteria set by us for identifying IFN-γ inducing peptides required adjusted counts in ELISPOT of >5 spots/well and at least 2/5 pigs having positive responses and therefore, some lowly reacting peptides could have been missed. As a matter of fact, nsp9 peptide aa 524-532 NYHWWVEHL examined by us overlapped with one of the peptides identified by Parida *et al.* [25] in positions 519-535 of that protein. In our case, individual responses in ELISPOT for that peptide were always below 5 counts. As evidenced in the present and other studies [22,23,24,25] individual variations attributable to pigs can have an influence on the results regardless of the approach taken. 

Next we analyzed SLA class I and class II haplotypes of pigs used in the experiment by PCR-based method using allele-group sequence-specific primers (PCR-SSP). The unresponsive pig in group I (pig 69) and one of the unresponsive group II (pig 64) were the only two animals being homozygous for SLA-I and SLA-II (Table 3). Although the number of examined pigs was not enough to establish clear correlations, it is interesting to note that SLA class I and class II homozygous pigs showed very poor IFN-γ to PRRSV suggesting that perhaps those animals had a very restricted panel of potentially recognizable epitopes. On the other hand, two of the high responders (pig 62 and 68) corresponded to a newly described haplotype and other high responders for genotype 1 or 2 vaccines (pigs 62, 66, 67, 70) share class I haplotype Lr-59.0 and class II haplotype Lr-0.27. Investigation of the relationship between haplotypes and response to PRRSV would merit further studies.

### 2.2. Evaluation of Peptide-Induced TGF-β and IL-10 Responses of PBMC

Since most peptides did not induce IFN-γ responses in immunized pigs, the question of whether or not those peptides have the capability to induce other cytokines such as TGF-β and IL-10 remained open. We decided to evaluate the ability of those peptides to induce other responses such as TGF-β and IL-10 release. Results did not support that any of the examined peptides induced TGF-β (data not shown); in contrast, some peptides induce IL-10 (Table 4). For this screening, it was considered positive those peptides inducing IL-10 release in PBMC cultures if two or more pigs of a group. Table 4 shows peptides that induce IL-10. Peptides that did not induce IL-10 (peptide aa 246-254 MAGINGQRF from nsp9, aa 1222-1230 YLPSYVLNC from nsp11, aa 3-11 FKLLAASGL from nsp9, and aa 2025-2033 IIIGGLHTL from nsp5) were no include in this table. Peptide aa 589-597 SLYKLLLEV of nsp2, which has been previously identified a IFN-γ inducer (Table 2), was not able to induce IL-10. This suggests that this is a peptide that needs more investigation to prove their participation in immunity and protection. In contrast, peptide aa 1929-1937 LLNEILPAV of nsp5 that induced IFN-γ, was also a strong inducer of IL-10. These are not necessarily contradictory facts. For example, it have been reported that in Hepatitis C virus the same peptide can induce Th1 and regulatory responses [26]. The rest of the peptides induce the production of IL-10, and only peptide, aa 1116-1124 KELAPHWPV of nsp11, induce IL-10 in a similar way that peptide aa 1929-1937 LLNEILPAV of nsp5. It would be interesting to test whether or not the IL-10 inducing peptides identified here contribute or not to the development of Treg. Interestingly, peptide SNLQLIYNLTLCELNGTDWL that had been previously reported as potential inducer of T regulatory cells (Treg) [27], was also IL-10 inducer. 

### 2.3. Inhibition of IFN-γ Responses by Peptides

The observation that some peptides were able to induce IL-10 responses leads us to examine if those peptides were able to inhibit IFN-γ responses induced by PHA. The experiments aimed to test the inhibition of IFN-γ responses showed that peptide aa 1116-1124 KELAPHWPV (a conserved peptide in nsp11) produced a significant reduction in the frequencies of IFN-γ secreting cells after PHA stimulation in 2/3 vaccinated and 2/3 unvaccinated pigs. Peptide aa 1929-1937 LLNEILPAV (a conserved peptide in nsp5) reduced the frequencies of PHA-induced IFN-γ secreting cells in 2/3 unvaccinated pigs and 1/3 vaccinated ones. The rest of the peptides tested produced inhibition in just one unvaccinated and one vaccinated pig, respectively (Table 5).

As show the results, when in the IFN-γ ELISPOT peptides were added to PBMC cultures stimulated with PHA inhibiting peptides were identified. Thus, two conserved peptides (aa 1116-1124 KELAPHWPV in nsp11 and aa 1929-1937 LLNEILPAV in nsp5) were able to inhibit significantly the frequencies of IFN-γ secreting cells in response to PHA regardless of whether the pigs were vaccinated or not. Therefore, this inhibition was not related to recall responses and suggest that this is probably an intrinsic property of most –or all- PRRSV strains. These results suggest that PRRSV may potentially inhibit IFN-γ responses, coincidentally, nsp11 is a protein known for their potential to inhibit also the expression of type I IFN [9].

## 3. Experimental Section

### 3.1. Experimental Design

Deduced amino acid sequences of polyproteins encoded by ORFs 1a and 1b of PRRSV genotype 1 [Genbank: M96262] were scanned for the detection of potential T-cell epitopes using bioinformatic prediction methods. Conservation of the predicted peptides was determined by comparison with a set of PRRSV sequences of genotype 1 and 2 isolates (n=40). Then, a set of the 18 best scoring conserved peptides (those peptides with one or none amino acid changes between genotype 1 and 2 isolates) and six non-conserved peptides (those peptides for which two or more amino acid changes between genotype 1 and 2 isolates were observed) were tested for their ability to induce IFN-γ responses in PBMC of immunized pigs. Additionally, the four best potential T-cell epitopes in nsp of prototype strains of genotype 1 and 2 -regardless of whether or not they were conserved- were tested. Also, the ability of the peptides for inducing IL-10 and TGF-β secretion in PBMC was examined. Peptides inducing IL-10 responses were further tested for their ability to inhibit IFN-γ responses in PBMC of vaccinated and unvaccinated pigs. 

**Table 4:** IL-10 levels (pg/mL) as determined by ELISA in cell culture supernatants of PBMC of PRRSV-vaccinated and unvaccinated pigs after stimulation with different peptides (10 µg/mL) of non-structural proteins of PRRSV. The table only shows peptides inducing IL-10 release in PBMC cultures of at least one pig.

Protein	aa position	Peptide sequence	Pigs of group I					Pigs of group II					Pigs of group III			
			66	67	68	69	70	61	62	63	64	65	71	72	73	74
nsp2	589-597	SLYKLLLEV	0	0	0	0	0	0	0	0	0	0	0	0	58	0
nsp2	1061-1069	GRFEFLPKM	70	0	0	0	37	0	0	0	0	0	0	0	0	0
nsp2	1141-1149	WLFAGVVLL	55	0	0	0	33	0	0	0	0	0	0	33	0	0
nsp3	1337-1345	YIWHFLLRL	0	0	49	40	0	0	0	0	0	0	0	0	42	0
nsp5	1929-1937	LLNEILPAV	82	0	50	0	33	0	0	0	0	0	0	41	34	0
nsp9	258-266	VLPGVLRLV	111	0	55	35	63	34	0	0	0	0	0	0	0	0
nsp10	716-724	IPYKPPRTV	0	0	51	0	35	0	0	0	0	0	0	0	43	0
nsp10	974-982	ITIDSSQGA	89	0	31	33	0	0	0	0	0	0	0	0	58	0
nsp11	1116-1124	KELAPHWPV	90	0	0	37	0	0	0	0	0	0	0	43	51	0
nsp11	1166-1174	GTPGVVSYY	43	0	0	44	36	0	0	34	0	0	0	0	0	0
ORF5	37-56	SNLQLIYNLTLCELNGTDWL	63	0	0	0	46	0	0	0	0	0	0	62	40	74
	Genotype 1 whole virus		218	0	0	50	48	0	0	0	0	0	0	34	72	33
	Genotype 2 whole virus		0	0	0	0	0	0	0	0	0	0	0	0	0	0
	PHA		88	117	86	60	52	45	130	137	54	73	375	63	96	40

**Table 5:** Effect of the addition of selected peptides to PHA-stimulated cultures on the frequency of IFN-γ secreting cells (per 5x10^4^ cells) of PBMC as determined in ELISPOT.

			**Group I**		**Group II**		**Group III**	
		**Pig no.**	**66**	**68**	**61**	**71**	**73**	**74**
		**PHA only ** **(10 μ** **g/ml)**	52 ± 3	49 ± 1	27 ± 3	73 ± 12	40 ± 5	50 ± 6
**Protein**	**aa position**	**Peptide sequence**	**PHA (10 μ** **g/mL) plus peptide (10 μ** **g/ml)**					
nsp2	1061–1069	GRFEFLPKM	54 ± 10	N.D.	N.D.	71 ± 12	N.D.	40 ± 4
nsp5	1929–1937	LLNEILPAV	35 ± 6*	39 ± 12	20 ± 14	70 ± 0	23 ± 8*	39 ± 14*
nsp9	258–266	VLPGVLRLV	44 ± 3	N.D.	N.D.	79 ± 4	N.D.	22 ± 4*
nsp11	1166–1174	GTPGVVSYY	35 ± 16*	N.D.	N.D.	83 ± 6	N.D.	38 ± 2
nsp11	1116–1124	KELAPHWPV	31 ± 0*	17 ± 7*	19 ± 5	67 ± 5	25 ± 7*	31 ± 6*
ORF5	37–56	SNLQLIYNLTLCELNGTDWL	28 ± 5*	45 ± 6	22 ± 4	66 ± 0	40 ± 3	32 ± 2*

N.D: Not determined; *p < 0.05 (Conover-Inman test)

### 3.2. Bioinformatic Prediction of Potential T-Cell Epitopes and Peptide Synthesis

Prediction of potential T-epitopes was carried out according to Diaz *et al.* [23] The predicted translation of ORFs 1a and 1b of Lelystad virus [genotype 1 prototype, Genbank: M96262] was used as a template for the screening. Briefly, prediction of nonamers binding to MHC-I was done with SYFPEITHI [28]. And for nonamers binding to MHC-II, the prediction was done using ProPred [29]. Peptides with the higher scores on SYFPEITHI and ProPred were further analyzed to assess the dissociation values (IC_50_ < 50 nM) using an online calculation tool [30]. Nonamers with the highest prediction scores and with the lowest dissociation values (IC_50_ < 50 nM) were chosen. The location of the predicted potential T-cell epitopes in the different non-structural proteins of PRRSV was determined using an alignment of polyproteins 1a and 1b of Lelystad virus and other eight genotype 1 isolates [Genbank: AY366525, AY588319, DQ489311, FJ349261, GU047344, GU047345, GU737264, JF802085, and M96262] and 31 genotype 2 sequences including prototype 2 strain VR-2332 [Genbank: AF176348, AY262352, AY545985, DQ459471, EF153486, EF635006, EU262603, EU360128, EU807840, EU860248, EU860249, EU880433, EU880438, FJ175687, GQ499193, GU232736,HQ401282, JF268682, JN626287, JN864948, JQ308798, JQ309822, JQ326271, JQ663556, JQ663568, JQ715697, JQ743666, JQ804986, NC_001961, and U87392]. 

Peptides located in conserved regions -at maximum one amino acid of difference between genotypes- that met the prediction criteria mentioned above were selected (nine peptides binding to MHC-I and nine binding to MHC-II). For four peptides having one amino acid of difference between genotypes, both variants were examined. 

Additionally, six best-scoring peptides with conservation between 80%–90% were included (two binding to MHC-I and four binding to MHC-II). Additionally, the four best scoring peptides binding to MHC-I in non-conserved regions for genotype 1 and genotype 2 PRRSV strains were selected. Also, a peptide previously reported to potentially induce regulatory T-cells (Tregs) was also included [27] Predicted peptides were produced by GL Biochem Ltd (Shangai, China) at a purity of ≥ 95% and adjusted to a concentration of 1mg/ml (master solution) in water or 50% methanol. Table 1 shows the peptides included in the present study.

### 3.3. Animals, Swine Leukocyte Antigen (SLA) Haplotyping and Vaccination

Four week-old piglets (n=14) were obtained from a PRRSV-free farm. Animals were confirmed to be sero-negative to PRRSV by ELISA at the beginning of the experiment (Herdchek X3, IDEXX Laboratories Inc). Animals were genotyped for their swine leukocyte antigen (SLA) haplotypes by running low-resolution PCR screening assays (PCR-SSP) on PBMC-derived genomic DNA as described by Essler *et al.* [31]. Animals were housed in the experimental farm of the Universitat Autònoma de Barcelona. At reception, pigs were ear-tagged and randomly (random numbers) distributed in three separated pens (designated as group I, n=5; group II, n=5 and; group III, n=4) where they were left to acclimatize for five days. Group I pigs were vaccinated with a commercial genotype 1 live attenuated PRRSV vaccine (Porcilis PRRS, Merck); group II was vaccinated with a genotype 2 live attenuated PRRSV vaccine (Ingelvac PRRS MLV, Boehringer-Ingelheim Vetmedica) and, group III was kept as an unvaccinated control. Vaccines were used according to their licensed dosage. 

### 3.4. Sampling and PBMC Isolation

Pigs were bled weekly in order to determine their serological status and to obtain peripheral blood mononuclear cells (PBMC) by means of a density-gradient centrifugation with Histopaque 1.077 (Sigma). PBMC were routinely adjusted to a density of 5×10^6^ PBMC/ml in RPMI 1640 medium (Sigma) supplemented with 10% fetal bovine serum (FBS), 2 mM L-glutamine (Sigma) and antibiotics. 

### 3.5. IFN-γ ELISPOT

The IFN-γ ELISPOT was carried out according to Diaz *et al.* [23] at 21, 49 and 56 dpv. Briefly, ELISA plates were coated overnight at 4 ºC with capture antibody P2G10 directed against porcine IFN-γ (BD Biosciences Pharmingen) diluted in 0.05 M carbonate-bicarbonate buffer pH 9.6. After washing with sterile PBS, 100 µl of PBMC were dispensed in plates (5 × 10^5^ per well) and were stimulated for 20 h with the genotype 1 or genotype 2 vaccine virus at a m.o.i. of 0.01 with the selected peptide at 10 μg/ml (as determined previously). The virus and peptides were added together. Un-stimulated PBMC and PHA-stimulated (10 μg/mL) cells were included as negative and positive controls, respectively. Each animal and assay was carried out in triplicate. After incubation, detection antibody P2C11 was dispensed into the wells and the ELISPOT assay was then revealed by the addition of insoluble TMB. In order to adjust antigen-speciﬁc frequencies of IFN-γ-producing cells (IFN-γ-SC), average counts of spots in un-stimulated wells were subtracted from average counts in antigen-stimulated wells. In the present case, results were expressed as the number of peptide-speciﬁc and PRRSV-speciﬁc IFN-γ-SC per 5x10^5^ PBMC to reflect actual counts on plates. In order to further assure the speciﬁcity of the tests, adjusted counts ≤5 spots were considered to be non-signiﬁcant or inconclusive.

### 3.6. Cytokine ELISAs

PBMC cultures (5x10^5^ PBMC/well) were stimulated with PHA, genotype 1 or genotype 2 PRRSV vaccine viruses at m.o.i. 0.1, peptides (10 µg/ml) or were mock-stimulated with culture medium for 20 h. For these experiments, a peptide that has been previously suggested to have the potential for inducing Tregs (SNLQLIYNLTLCELNGTDWL) was also included [27]. Then, cell culture supernatants were collected and frozen at -80°C until used. TGF-β was quantified in the supernatants using a multispecies TGF-β kit and IL-10 was quantified using an in-house ELISA developed using appropriate antibody pairs (Invitrogen). In each plate a two-fold dilution series of cytokine standards provided by the manufacturer were included (from 2000 pg/mL to 31.25 pg/mL for IL-10 and from 4000 pg/ml to 62.5 pg/mL for TGF- β) plus a blank (0 pg/mL). 

To assure the specificity of the results, the cut-off of the ELISAs was calculated as the mean optical density plus three standard deviations of the blank sample. This value was compared with the average optical densities of the standard dilution series to determine the lower amount of cytokine that could be determined with precision. Then, for samples which optical densities were above the cut-off, cytokine concentrations were calculated using the correlation formula obtained from the cytokine standards.

### 3.7. Inhibition of IFN-γ Responses by Peptides

Peptides that did not induce IFN-γ and induced IL-10 response were further tested for their potential to inhibit IFN-γ responses. Two pigs of group I, one pig of group II and three pigs of group III were included. Inhibition of IFN-γ was examined by adding 10 µg/ml of the selected peptides to PHA-stimulated cells (5 × 10^4^; triplicates). IFN-γ production was evaluated by ELISPOT as above. 

### 3.8. Statistical Analysis

Data were analyzed using the non-parametric Kruskal-Wallis and Conover-Inman tests performed with StatsDirect v2.7.8 (StatsDirect Ltd, UK). Graphic images were constructed with GraphPad Prism software v5.

## 4. Conclusions

The present work describes one peptide in nsp2 of PRRSV able to induce IFN-γ. In addition, we describe that nsp may contain regions resulting in IL-10 induction and, in some peptides, inhibition of IFN-γ responses. These data are relevant for the development of future vaccines.
